# Improving access to clinical practice guidelines with an interactive graphical interface using an iconic language

**DOI:** 10.1186/1472-6947-14-77

**Published:** 2014-08-26

**Authors:** Suzanne Pereira, Sylvain Hassler, Saliha Hamek, César Boog, Nicolas Leroy, Marie-Catherine Beuscart-Zéphir, Madeleine Favre, Alain Venot, Catherine Duclos, Jean-Baptiste Lamy

**Affiliations:** 1 VIDAL, 21 rue Camille Desmoulins, 92789 Issy les Moulineaux, France; 2 INSERM CIC-IT/Evalab, Université Lille Nord, Lille, France; 3 UDSL EA 2694, CHU Lille, 59000 Lille, France; 4 Université Paris Descartes, Faculté de Médecine, Département de Médecine Générale, Paris, France; 5, Société de Formation Thérapeutique du Généraliste (SFTG), Paris, France; 6, Laboratoire d’informatique médicale et d’ingénierie des connaissances en e-santé (LIMICS), Université Paris 13, Sorbonne Paris Cité, INSERM UMRS 1142, Université Paris 6, 74 rue Marcel Cachin, 93017 Bobigny, France

**Keywords:** Practice guidelines as topic, User-computer interface, Computer graphics, Iconic language

## Abstract

**Background:**

Clinical practice guidelines are useful for physicians, and guidelines are available on the Internet from various websites such as Vidal Recos. However, these guidelines are long and difficult to read, especially during consultation. Similar difficulties have been encountered with drug summaries of product characteristics. In a previous work, we have proposed an iconic language (called VCM, for Visualization of Concepts in Medicine) for representing patient conditions, treatments and laboratory tests, and we have used these icons to design a user interface that graphically indexes summaries of product characteristics. In the current study, our objective was to design and evaluate an iconic user interface for the consultation of clinical practice guidelines by physicians.

**Methods:**

Focus groups of physicians were set up to identify the difficulties encountered when reading guidelines. Icons were integrated into Vidal Recos, taking human factors into account. The resulting interface includes a graphical summary and an iconic indexation of the guideline. The new interface was evaluated. We compared the response times and the number of errors recorded when physicians answered questions about two clinical scenarios using the interactive iconic interface or a textual interface. Users’ perceived usability was evaluated with the System Usability Scale.

**Results:**

The main difficulties encountered by physicians when reading guidelines were obtaining an overview and finding recommendations for patients corresponding to “particular cases”. We designed a graphical interface for guideline consultation, using icons to identify particular cases and providing a graphical summary of the icons organized by anatomy and etiology. The evaluation showed that physicians gave clinical responses more rapidly with the iconic interface than the textual interface (25.2 seconds versus 45.6, p < 0.05). The physicians appreciated the new interface, and the System Usability Scale score value was 75 (between good and excellent).

**Conclusion:**

An interactive iconic interface can provide physicians with an overview of clinical practice guidelines, and can decrease the time required to access the content of such guidelines.

## Background

Clinical practice guidelines (CPGs) are “systematically developed statement to assist practitioner and patient decisions about appropriate health care for specific clinical circumstances” [1]. They provide physicians with helpful recommendations for the diagnosis and treatment of diseases [2]. However it has been shown that physicians often do not follow guidelines, a phenomenon known as “clinical inertia” [3]. Various approaches have been proposed to increase guideline dissemination and adoption [4], one of them being computerized guidelines. However, even these computerized guidelines obtained limited results and clinicians’ perception of them is a key factor for success [5]. Most attempts to improve the electronic access of physicians to CPGs have focused on the computer execution of guidelines [6] rather than on the visualization and presentation of CPGs. Applications may allow parts of CPGs to be extracted or automatic alerts to be raised, but this requires structured and coded patient data. However, structured patient data may be unavailable, for example when i) a physician does not enter patient data or enters data as non-computer-interpretable free text, which occurs often [7], ii) data coding is unreliable, also a frequent problem [8], or iii) a physician reads a guideline for educational purposes rather than searching for information relating to a given patient. In these situations, access to the full CPG remains necessary.

Almost all CPGs are provided in a textual format, sometimes including a few algorithms and tables. However, reading textual CPGs can be tedious and very time-consuming. One of the ten grand challenges in clinical decision support is to improve human computer interface [9], and T. Sinuff et al. [10] have shown that physicians and nurses prefer algorithms, tables or graphs rather than text and sentences. S.E. Rosenbaum [11] showed that navigating through the Cochrane Library, and reading the reviews it proposes, was difficult for physicians. Time availability is limited during consultations, and a physician may give up the search for an answer to a clinical question after only a short time, generally within two minutes [12].

Similar problems are encountered when reading other medical texts, and icons and pictograms have been proposed as a solution to facilitate access to medical texts or data. Many pictograms have been proposed to convey information about drug prescriptions to patients [13,14]. For health professionals, Uval Med [15] offers a graphical presentation and definition of various diseases. The Visual Language system (VLsys) [16] defines compositional icons for representing medical concepts, and animated glyphs for representing verbs relating those concepts. VLsys aims to facilitate the identification of concepts and the discovery of new information, for example from a list of Pubmed’s search results. In Stabilis 3 [17], icons are used to present the stability and compatibility of injectable drugs in a dedicated database. Specific icons have also been used to represent the patient’s sex, size and weight [18]. In a previous study on drug summaries of product characteristics (SPCs) [19,20], we proposed the Visualization of Concepts in Medicine (VCM), a language that uses icons to represent medical concepts including symptoms, disorders, physiological states (such as age class or pregnancy), risk and history of disorders, drug and non-drug treatments, lab tests and follow-up procedures. VCM icons were used for representing contraindications of drugs, cautions for use or adverse effects. We designed a graphical user interface called “Mister VCM” [20] that makes use of these icons, organizing them by anatomy and etiology according to schematic characters. This approach has yielded promising results as we found that VCM and “Mister VCM” decreased both the time required to find a piece of information and the risk of error. More recently, VCM icons have been used for facilitating information retrieval in a search engine targeting medical guidelines [21].

**Figure 1:**
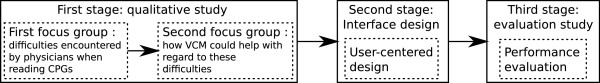
Flowchart of the three stages of the work presented.

The objectives of the current work were to design an interactive user interface facilitating the consultation of CPGs by physicians using the VCM iconic language, and to evaluate this interface in terms of its performance and user-perceived usability. We used a user-centered design approach [22,23], in three stages (see Figure 1). The first stage was a qualitative study based on focus groups involving physicians, focusing on their difficulties with CPGs and how VCM could help them. The second stage was the design of the user interface, taking into account the results of the previous study. The third stage was the evaluation of the performance of a prototype iconic interface for consulting CPGs.

We assembled a group of partners in the L3IM project (*Langages Iconiques et Interfaces Interactives en Médecine*, Iconic Languages and Interactive Interface in Medicine), including experts from various backgrounds (physicians, pharmacists, ergonomists, computer scientists and medical information scientists) and held a discussion and brainstorming session. The partners included three academic partners specialized in medical informatics: (LIM&BIO, *Laboratoire d’Informatique Médicale et de Bioinformatique*, Laboratory of Medical Informatics and Bioinformatics, now LIMICS, see affiliations, and CISMeF team, *Catalogue et Index des Sites Médicaux de langue Française*, Catalog and Index of French-language Health Internet resources) and medical devices/informatics-related human factors (CIC IT/Evalab team), an associative partner involved in continued education for physicians (SFTG, *Societé de Formation Thérapeutique du Généraliste*, The Society for Therapeutic Education for General Practitioners), and several industrial partners including the editor of a drug database and a website providing prescription guidance (Vidal), a vendor of hospital electronic patient records (McKesson France), a vendor of shared electronic patient records (Santeos) and a vendor of software for medical offices (Silk Informatique).

In this article, we briefly introduce Vidal Recos, a website presenting CPGs in textual format, which we used as the starting point for this study, and the VCM iconic language. We then describe the qualitative study and the difficulties encountered by physicians that it identified, together with the recommendations made by ergonomists for the design of the iconic interface. We then describe the design of the prototype iconic interface, based on VCM and Vidal Recos, and the resulting interface, followed by the evaluation study and its results. Finally, these results are discussed in the context of various existing approaches for presenting guidelines.

## Materials

### Vidal Recos

Vidal Recos (http://www.vidal.fr/recommandations/index/) is a collection of French CPGs available online. The website aims to provide an interface tool for medical recommendations during consultations based on the CPGs produced by the French National Health Authority (HAS, *Haute Autorité de Santé*) or by learned societies. Vidal Recos is updated monthly and used by many health professionals, including those working in hospitals. Vidal Recos currently includes 165 CPGs, each of them targeting a specific disorder or patient condition and covering the most frequent case scenarios. Each guideline follows the same structure with the following sub-titles: “Definition of the disorder”, “Diagnosis”, “Which patients should be treated?”, “Care objectives”, “Medical care”, “Treatments” (including a list of drugs) and “Bibliographic references”. The “Medical care” chapter includes a decision tree, a list of particular cases, considerations when evaluating care and advice for patients (a French example of the recommendation for multiple sclerosis can be seen at: http://www.vidalrecos.fr/pages/reco.php?idfiche=2712).

### The VCM iconic language

The VCM language [19] proposes icons for representing the main clinical conditions of patients (including symptoms, disorders and physiological state (for example, age class or pregnancy)), risk and history of disorders, use of drug and non-drug treatments, laboratory tests and follow-up procedures. It aims to complement medical texts (rather than to replace them) by highlighting pieces of text or helping physicians to find the desired part of the text. VCM includes a set of graphical primitive (shapes, pictograms and colors), and uses graphical language to combine these elements and create icons.

**Figure 2:**
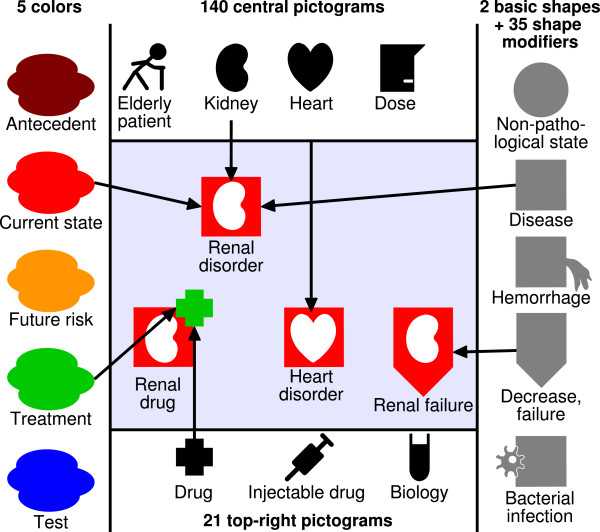
**Examples of VCM icons created by combining shapes, pictograms and colors.** The simple “renal disorder” icon is created by assembling the red color (current state), the square (disease) and the kidney pictogram. It can then be further modified to create the “drug for renal disorder” icon (by adding a green cross top-right pictogram (meaning drug treatment)), or the “renal failure” icon (by adding a shape modifier showing a downward arrow (meaning a decrease or failure)).

A VCM icon can be described by a color, a basic shape and set of shape modifiers, a central pictogram, a top-right color and one or two top-right pictograms. Figure 2 illustrates the graphical combinations of these elements. A simple icon can be created by combining i) a color indicating the temporal aspect of the icon: red for current states of the patient, orange for risks of future states and brown for past states (such as antecedents or history), ii) a basic shape: a circle for physiological states (*i.e.* normal states) or a square for pathological states (disorders or symptoms), iii) a central white pictogram indicating the anatomico-functional location (*e.g.* a heart pictogram indicating both the heart and cardiac function) or the patient characteristic (such as pregnancy) involved and iv) zero, one or several shape modifiers indicating general types of disorders and morphologies (for example, a small bacterium for bacterial infection or a downward arrow for deficiency) or “transverse” anatomical structures present in most organs (such as blood vessels).

Icons for treatments or follow-up procedures are created by taking the corresponding icon for the disorder treated or the risk of disorder monitored, and adding a top-right pictogram in green (treatment) or blue (follow-up procedure). The shape of the top-right pictogram indicates the type of treatment (*e.g.* drug treatment, oral drug or surgery) or follow-up procedure (including laboratory tests and medical imaging). A second top-right pictogram can be added to represent health professionals or medical documents. For example, the cardiologist icon is created by adding the health professional top-right pictogram to the cardiac disorder icon.

**Figure 3:**
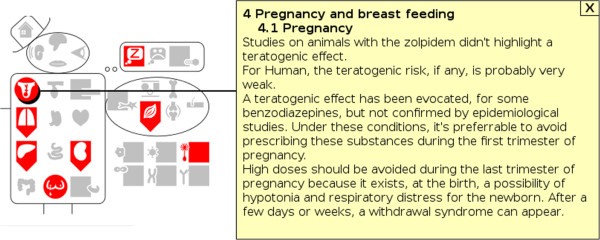
**Screenshot of “Mister VCM” displaying the contraindications of a soporific drug.** The user has clicked on the “pregnancy” icon, and the corresponding text is shown.

“Mister VCM” [20] is a graphical user interface that provides a visual summary of a set of VCM icons (see Figure 3). The interface organizes icons according to anatomy and etiology, using a simplified anatomical schema. “Mister VCM” is interactive and can display additional information when the user clicks on an icon.

More information about VCM can be found at the VCM website (http://vcm.univ-paris13.fr/) and in the article describing VCM [19]. The semantics of VCM has recently been formalized through the use of an ontology [24], and the main categories of VCM are consistent with the UMLS semantic network and medical terminologies, including SNOMED CT in particular. The main medical concepts (anatomical structures, biological functions, etiologies,...) are present in VCM. Similarly, the UMLS semantic network distinguishes physiological and pathological functions, normal and abnormal anatomical structures, corresponding to the VCM circle and square associated with physiological and pathological states, respectively.

## Qualitative study for user-centered design

### Study design

We organized focus groups involving physicians. Focus group studies are qualitative studies essentially corresponding to a collective interview. Through group dynamics, it is possible to explore various point of view, to answer “Why?” and “How?” questions, and to identify the problems encountered by professionals. Two focus group sessions were organized, involving the same participants. The first session focused on the difficulties encountered by physicians in the consultation of CPGs, and the second session focused on the ways in which an iconic language, such as VCM could facilitate the reading of CPGs.

### Participants

Eight General Practitioners’ (GPs) were recruited *via* a French association providing GPs with ongoing training. Four ergonomists were responsible for running the focus groups.

### Procedures

The first focus group (one half day) aimed to identify the difficulties faced by physicians trying to consult CPGs. Physicians were given two CPGs: one in paper format (CPG for acne, 14 pages) and one in Vidal Reco electronic format (hypothyroidism in adults). These CPGs were chosen because they target frequent disorders and belong to different medical specialties. The physicians were initially asked to read the paper CPG, to annotate it with highlighter pens and Post-It notes, and to solve a medical scenario using the CPG (see Table 1). They were then asked about (a) the difficulties they encountered when trying to consult the document, (b) situations in which they search for information in similar CPGs, and (c) the solution they would propose for facilitating access to the recommendations of CPG. A similar procedure was then followed for the electronic CPG, with the physicians being asked to “think aloud” when using the computer. Finally, at the end of the first session, physicians were introduced to VCM and provided with training software.

**Table 1:** Examples of scenarios used during the qualitative (first) study and the evaluation (second) study (translated from French)

**#**	**Usages**	**Disorders involved**	**Scenarios**
1	Qualitative study, first focus group, paper CPG	Acne	A 35-year-old female patient in the 24th week of pregnancy comes to see you for inflammatory acne (localized form). She comes back a few days later because of intolerance (burns) to the treatment you prescribed her (adaptalen). You go to the Vidal Recos website to obtain information relating to: therapeutic management, how to reassure the patient regarding this adverse effect, precautions applying to this situation.
2	Qualitative study, first focus group, electronic CPG	Hypothyroidism	One of your patients, a 70-year-old man with a history of coronary disease, comes to his consultation with biological test results, so that you can determine the most appropriate prescription for him. You are following this patient for hypothyroidism. His current TSH (thyroid-stimulating hormone) level is 4. What approach do you adopt?
3	Evaluation study	Hypertension	A 32-year-old female patient in the seventh month of pregnancy comes to see you. It is her second pregnancy and she has a blood pressure of 150/80. Her blood pressure measured at home 15 days ago was 145/80. This patient has been monitoring her blood pressure since her first pregnancy because she has hypertension, and she has been treated with a drug (she forgets its name). You wish to see the recent recommendations for managing hypertension in pregnant women, particularly as concerns the drugs that you can prescribe her.

The second focus group (one half day) took place 15 days later. We first asked the physicians for their opinions about VCM, following their use of the training program, and provided them with a short (20 minutes) training session to “refresh” their memory. We then presented them with a new paper CPG (osteoporosis, 23 pages). As the CPG was long, we set up two groups of four GPs. One group received the following part of the CPG: decision tree, particular cases, advice to the patient and description of the disease. The other group received the decision tree, criteria for treatment, aim of treatment and treatments. We asked physicians about how VCM icons could help them to use the CPGs. We provide them with stickers showing VCM icons and asked them to tag the CPG, and with larger stickers for use on a paperboard during the general discussion. GPs, working in pairs, were asked to choose the VCM icons representing the significant information contained in the CPG and to produce a synthesis of the CPG, using VCM icons mixed with short text, tables or schemas. The resulting syntheses were then shared with the other members of the group and discussed by the participants.

### Variables

During the two focus groups, we collected (a) the annotated paper CPGs and the syntheses, (b) the notes taken during the solving of the scenarios, (c) a recording of what the physicians said when “thinking aloud” during the use of the electronic CPG, (d) the general discussion. For (c) and (d), tape recordings were obtained and transcribed for analysis.

### Results

Table 2 summarizes the types of information and sections of the CPG highlighted or annotated by the physician during the first focus group. The “particular cases” section was by far the most annotated section. The difficulties identified during the first focus group are shown in Table 3. They included, in particular, the absence of an appropriate summary and difficulties identifying the right particular cases. During the second focus group several suggestions for the integration of VCM into Vidal Recos emerged. These suggestions were noted and synthesized by ergonomists, who then provided several recommendations, as shown in Table 4.

**Table 2:** The type of information highlighted by the eight physicians during the first focus group, and the sections of the CPG they annotated

**Types of information highlighted**	**Number of physicians**
Treatment envisaged	5
Contraindication	4
Initial treatment	2
Advice for the patient	2
Physiopathology	1
Epidemiology	1
Diagnosis	1
Drug indication	1
Risk	1
**Annotated sections of the CPG**	**Number of physicians**
Particular cases	7
Decision tree	3
Treatment	3
Diagnostic	2
Advice for patient	2
Definition of the disorder	1

**Table 3:** The difficulties relating to the consultation of CPGs by physicians, as identified during the first focus group

**#**	**Difficulties**	**GP quotes**
1	Summaries are frequently missing, making it difficult to obtain a clear general overview of a CPG	“This is good for research or education but not for medical practice because we have to search for the right information; a synthetic card would be welcome”
2	It is difficult to find particular cases and exceptions; tables of contents and decision trees are not the most suitable approach for this	“This is annoying, we cannot find particular cases in the tree”
3	It is difficult to relate the nodes on decision trees, or boxes in diagrams, to their corresponding text in the CPG, even in the electronic CPGs (which did not provide links for this purpose)	“You must browse several pages to find what you seek”
4	CPG texts are long, and clinically important terms are difficult to identify as they are not highlighted	
5	CPGs can include many ambiguous sentences	

**Table 4:** The recommendations for the integration of VCM in Vidal Recos, formulated during the second focus group

**#**	**Recommendations**
1	The disorder targeted by the CPG should be represented by a VCM icon at the beginning of the CPG
2	Summaries should be proposed, including VCM icons or a “Mister VCM”
3	The diagnostic elements, the particular cases, the treatments and the follow-up procedures should be identified by VCM icons
4	VCM icons should be inserted in decision trees, and also used to distinguish the trees when a CPG includes several trees
5	VCM icons should be clickable links to the corresponding text in the CPGs

We also collected the comments of physicians about VCM icons at the beginning of the second focus group. The VCM icons were generally well appreciated. Several pictograms, such as those for the thyroid gland, were considered unclear. Another more general problem concerned the icons for treatment involving arrows. As VCM icons for treatments are based on the icon of the treated disorder, the direction of the arrow corresponds to the disorder rather than the treatment. For example, hypothyroidism is represented by the thyroid pictogram and a downward arrow. Consequently, thyroid hormone substitutes are represented by the thyroid pictogram, the downward arrow and the green cross. Physicians also suggested new icons for specific disorders (for pleural effusion, zona, catheter infection and extreme wasting), and new elements of information that could be added to the existing icons (distinguishing between chronic and acute clinical conditions, and between stable and unstable conditions).

## Design of the graphical user interface

### Methods

Several suggestions for the integration of VCM icons into Vidal Reco emerged during the user-centered study, and all were considered. However, we decided to focus on only a few suggestions, as the ergonomists warned us to avoid having too many icons on the screen, particularly if they have different usages. We chose to focus on particular cases, because the treatment of particular cases was identified as a problem by several GPs (during the focus group, see Table 3 item #2) and the particular cases section was the section with the most annotations in the qualitative study (see Table 2). Moreover, VCM seemed to be suitable for the representation of these cases. We therefore suggested the identification of particular cases by icons in the text (recommendation #3 in Table 4), and the addition of a “Mister VCM” summarizing these cases (#2) with clickable icons (#5). It was technically difficult to add icons to the decision tree.

Several pictograms were redesigned in accordance with the suggestions made by the GPs. However, it was not possible to take other suggestions into account without major changes to VCM, which we wished to avoid. The choice to represent treatments as the treated disorder was much debated during the initial design of VCM, and this approach was adopted because many treatments are expressed as “anti-(a given disorder)” (although this was not the case for hypothyroidism treatments). The distinction between chronic and acute and between stable and unstable clinical conditions would be interesting, but would make the icons more complex.

A draft preliminary prototype of the graphical user interface was implemented. Five physicians performed usability tests on this interactive prototype. The physicians were given specific instructions, including a “think-aloud” protocol, and their activities were recorded with a camera and a microphone while they carried out the tasks. Five tasks were considered, the three first of which involved the icons in “Mister VCM”: (1) displaying the label associated with an icon on “Mister VCM”, (2) displaying the list of paragraphs associated with an icon, (3) displaying the first paragraph associated with the icon, (4) displaying the label associated with an icon in the margin of the text, and (5) highlighting a text portion by clicking on an icon. For each task, the time required and the status (achieved or not) were recorded. A debriefing session was held after completion of the tests. Ergonomists analyzed the problems encountered by physicians during the usability tests, using the MORAE^®;^ software and following the recommendations of ISO norms 13407 (Human-centred design processes for interactive systems) and 25062 (Common Industry Format for usability test reports). Then they formulated recommendations for improving the interface. The only major recommendation was that “Mister VCM” was not visible enough in the preliminary prototype. We therefore made it more visible in the final prototype.

### The graphical user interface for consulting CPGs

**Figure 4:**
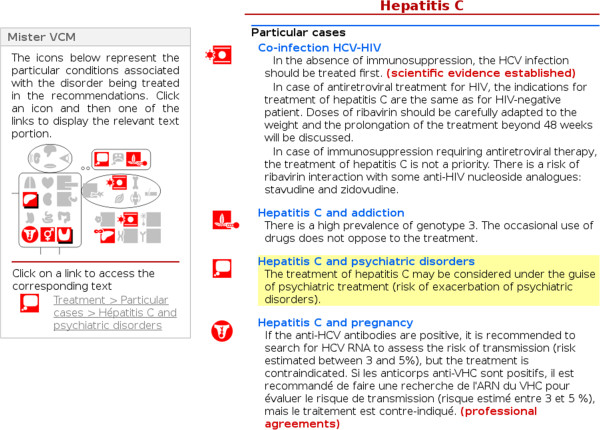
**Screenshot of the Vidal Recos prototype integrating VCM, displaying a CPG related to hepatitis C.** In this screen shot, the physician has clicked on the “psychiatric disorder” icon on “Mister VCM” (on the left of the screen), and the corresponding paragraph has been searched for and is highlighted in yellow in the text (on the right).

We designed a prototype of the Vidal Recos’ interface integrating VCM icons and “Mister VCM”. In this prototype, VCM was used to highlight particular cases, such as pregnant women, children, diabetic patients or patients with renal failure. Figure 4 shows a screenshot of the prototype. VCM icons were added in the left margin of the CPG text. These icons were used to tag the various additional patient conditions associated with a given paragraph of the CPG, to help physicians to find the answer to questions like “what is recommended for a patient with this condition?”. An example of such question when reading a CPG related to hypertension is “what is recommended for a hypertensive patient who is also diabetic?”. The icons are interactive: when a physician places the mouse over an icon, the corresponding part of the text (possibly one or several sentences or paragraphs) is highlighted with a yellow background.

A “Mister VCM” was added to the left column of the interface, bringing together the icons for the various patient conditions present in the CPG and organizing them by anatomy (*e.g.* heart, kidney and lung) and etiology (*e.g.* infection and tumor). When a physician clicks on one of these icons, a short list is displayed at the bottom of “Mister VCM”. This list includes one item per paragraph or sentence relating to the VCM icon selected by the physician (each item consisting of an icon and a textual label). A second click on one of the items automatically scrolls the page to the corresponding paragraph and highlights the text. The left column containing “Mister VCM” is always visible on the screen, making it possible for the physician to click on another icon without having to scroll to the top of the page. The “Mister VCM” allows the physician to go quickly to the part of the CPG relating to a given patient condition.

An example of the final interface can be consulted online [25]. This interface was developed after the work presented here (after both design and evaluation) and it proposes the same features, but with a graphical chart slightly different from Figure 4.

### Graphical user interface for annotating CPGs

The icons present in the margin or in the “Mister VCM” were manually selected by medical experts. A specific annotation tool, based on the Scenarii Open Source software (http://scenari-platform.org), was designed to help with CPG annotations. This tool can be used to select the icons to be associated with CPG paragraphs, *via* several different methods. It provides a list of the most frequently used icons, a list of the recently used icons, a textual search box (*e.g.* type “renal failure” to get the renal failure icon), and a module for creating icons by combining pictograms, shapes and colors (*e.g.* combine the “kidney” pictogram with the “failure” shape to get the renal failure icon). Icons associated with a paragraph can then be included in “Mister VCM”.

## Evaluation of the graphical user interface

### Study design

The prototype interactive iconic interface was evaluated by comparing it to the initial interface of Vidal Recos (a textual interface without VCM). We evaluated performances (response time and response accuracy) for each interface, and the perceived usability of the new interface.

We designed two medicaly-validated scenarios, each describing a specific patient, his history and his health problems, based on real cases.These scenarios were different from those previously used in the focus groups. The first scenario was based on the hypertension CPG and involved a hypertensive pregnant woman (see Table 1). The second was based on the nephritic colic CPG. These scenarios were chosen because they involved two disorders frequently encountered by GPs and belonging to different medical specialties (cardiology and infectious diseases). They also involved particular cases from the CPGs (such as pregnancy), which the physicians identified as a difficult point during the focus group. Each scenario included a description of the patient and a question related to treatment prescription (“What therapeutic treatment do you propose for this patient?”). The scenarios were unambiguous and lead to a clear “undebatable” answer in the CPGs. For each scenario, we selected the CPG paragraph considered to answer the question most appropriately. It was defined by two physicians, both having selected the same paragraph. The “correct answer” to the scenario was considered to be the treatment recommended in that paragraph.

### Participants

We recruited 20 physicians: 10 GPs from a French association that performs continuing education, and 10 hospital physicians from Rouen Hospital. Each physician was provided with a 10-minute introduction to Vidal Recos, the interface and VCM. The VCM presentation focused on the types of icons the physicians would be likely to encounter during the evaluation, due to limited time for the explanation.

### Procedures

Physicians were asked to search for the appropriate treatment using the corresponding CPG. CPGs were displayed with the Vidal Recos graphical interface, either with the interactive iconic interface or the textual interface. Each physician analyzed the two scenarios, one with the interactive iconic interface and one with the textual interface. Half the physicians began with one interface and the other half began with the other. The order of the two scenarios, the interface and the type of physician were randomly assigned to four groups as follows: i) five GPs tested the first scenario with the textual interface and then the second scenario with the interactive iconic interface, ii) five GPs tested the first scenario with the interactive iconic interface and then the second scenario with the textual interface, iii) five hospital physicians tested the second scenario with the textual interface and then the first scenario with the interactive iconic interface and iv) five hospital physicians tested the second scenario with the interactive iconic interface and then the first scenario with the textual interface.

### Variables

During the performance evaluation, we recorded the response time (the time taken by the physician to find the appropriate treatment in Vidal Recos) and the response accuracy (whether the information obtained corresponded to the CPG paragraph containing the appropriate answer, as defined for each scenario). Perceived usability was measured by asking each physician to complete a System Usability Scale (SUS) questionnaire [26] after the performance evaluation.

### Statistical analysis

We considered the type of interface (interactive iconic *vs.* textual), physician (n = 20) and scenario (n = 2) as factors when comparing response times. A Shapiro-Wilk normality test showed that the response times were not normally distributed (p = 1.7×10 ^-7^), so a logarithmic transformation was applied to response time. Transformed response times were then found normally distributed according to the Shapiro-Wilk test (p = 0.31). A Bartlett test showed that the variances were homogeneous (p = 0.11), and ANOVA was used to investigate the effect of the type of interface, physician, scenario and their interactions on response time. A Fisher’s exact test was used to compare the number of errors depending on the type of interface. Significance thresholds were set at *α*=5*%*. Data were analyzed with R software version 2.14.2 [27].

### Results of the performance evaluation

There were 40 responses in total; two physicians made errors with the textual interface and no errors were made with the interactive iconic interface. This difference was not significant (Fisher’s exact test, p = 0.49).

Physicians were asked to find the appropriate treatment using the Vidal Recos interface, with either an interactive iconic interface or a textual interface. When using the interactive iconic interface, all physicians spontaneously used “Mister VCM” to search the CPG text. The average response time was 45.6 seconds (95% CI: 29.1 − 62.0) with the textual interface, and 25.2 seconds (95% CI: 20.5 − 29.8) with the interactive iconic interface. ANOVA (performed after logarithmic transformation, degrees of freedom: 32) showed that physicians responded significantly faster when using VCM (p = 0.040, F = 4.591). The mean response time in trials with the textual interface was 1.8 times that with the interactive iconic interface. Other factors (physician and scenario) were not significantly related to response time (p = 0.66 and p = 0.99, respectively), and there was no significant interaction between the factors.

### Results of the perceived usability evaluation

**Figure 5:**
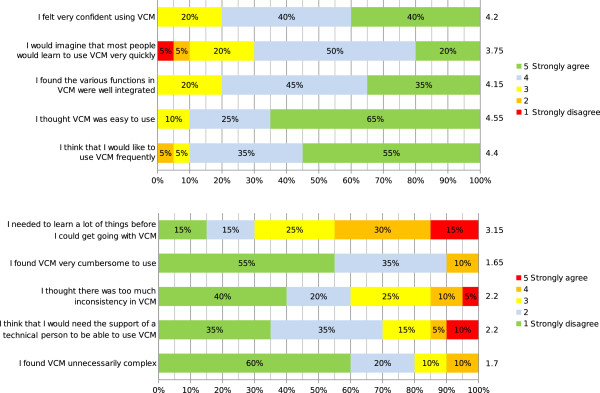
**Results of the SUS questionnaire.** The number on the right of each bar indicates the mean score for the question (ranging from 1 to 5).

**Figure 6:**
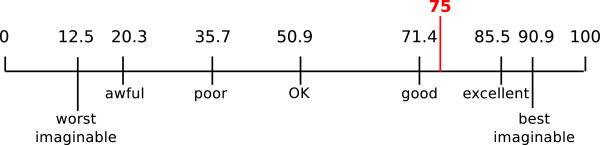
**SUS score obtained.** The seven adjectives shown at the bottom and the corresponding SUS scores are those proposed by A. Bangor et al. [28].

Figure 5 shows the results obtained for each of the ten questions in the SUS questionnaire. The mean SUS score was 75 for the interactive iconic interface with VCM (Figure 6), corresponding to a positive appreciation between “good” and “excellent” [28]. Most physicians found VCM easy or very easy to use, and 90% said they would like to use it frequently. However, most felt that they would need additional training before using VCM.

The physicians interviewed said that they found VCM intuitive to use and were convinced that they could gain time by using it. In particular, they appreciated the “Mister VCM” icons for accessing particular cases rapidly and the icons tagging CPG paragraphs that dealt with specific patient conditions. However, the physicians felt that “Mister VCM” was not sufficiently visible in Vidal Recos.

## Discussion

We have presented the integration of VCM (a medical iconic language) into Vidal Recos, a website for the consultation of CPGs by physicians. We identified current CPG difficulties encountered by physicians, designed a prototype interface taking into account human factors and evaluated the performance of the interface and physician perceived usability when using it. The proposed interface uses VCM icons to tag patient conditions in CPG text and “Mister VCM” to provide an interactive summary of the particular cases discussed in the CPG, organized by anatomy and etiology.

The performance evaluation showed promising results: physicians using our interactive iconic interface found recommendations that apply to a specific patient significantly faster than with a traditional textual interface. The difference in response times (1.8 times longer with the textual interface) was similar to that observed for a comparison with the use of drug SPCs [19,20]. The number of errors was lower when using the interactive iconic interface (0 *vs.* 2). This difference is not significant, possibly due to the small number of physicians and scenarios in the study.

The perceived usability evaluation showed that physicians found VCM easy to use and were enthusiastic about it. They suggested that they would need additional training before using VCM which is not surprising given the short duration of the training session for this study (about 10 minutes).

The evaluations compared the new interactive iconic interface *vs.* a textual interface. The difference observed between the two interface could be related to the presence of VCM icons, or to the interactive aspect of the “Mister VCM” interface. The physicians’ comments suggested that interactivity was an important factor: the interactive “Mister VCM” was used extensively by physicians and they found it very convenient.

The evaluation did not occur in a “real” clinical situation. Instead, it was an “in vitro” evaluation, and this constitutes one of the limitations of the study. Another limitation was the focus of the performance evaluation on two scenarios extracted from two guidelines, one for hypertension and the other for nephritic colic. However, the VCM language provides icons for many other clinical conditions that can be applied to a wide range of guideline. The iconic interface could therefore be used for many other guidelines, and 165 guidelines are implemented in the commercial Vidal Reco product released after the study. A larger evaluation involving more physicians and guidelines, possibly in clinical situations, would be of particular interest, to determine whether the impact of the icons depends on the medical specialty or not.

The fact that physicians have to learn the VCM language is one of the main limitations of the proposed interface. In a previous study, we found that a learning time of four to six hours was sufficient [19]. However, it has been shown with VLsys [16] that iconic languages can be used without formal training, with progressive learning “on the fly”, from tooltips and animations. It would therefore be interesting to evaluate the impact of various VCM learning times, and to determine whether the initial training could be replaced by “on the fly” learning. Color-blind physicians may encounter difficulties when viewing VCM icons due to the use of red and green color-coding. However, VCM icons are computer-generated and it should be possible to modify the default colors according to user preferences (*e.g.* using gray and black rather than red and green for color-blind people).

The impact of visual data display has been widely studied in the medical field [29]. Most of these studies focused on quantitative data (such as the chances of survival or adverse drug event frequency), and compared various formats including bar charts, tables and pictographs. We used similar evaluation methods, although our study involved the presentation of medical knowledge rather than quantitative data. Three aspects are particularly important when evaluating a visual display: the user’s comprehension (did the user understand the data displayed well?), choice (did the user make the correct choice in a given situation, when using the visual display?) and the user’s preference (which visual displays did the user prefer?). These three aspects correspond approximately to the criteria used here to evaluate the iconic interface: response time, to measure the physician’s comprehension (as proposed by Hildon [29]), number of errors, corresponding to the “choice” aspect, and perceived usability, which provides a representation of user preferences as it measures the user’s subjective appreciation.

W. Aigner et al. reviewed the usual methods for visualizing CPGs [30]. They distinguished “model-centric” approaches, where the meaning of CPG text is extracted and no longer related to the original text, and “document-centric” approaches, where the text of the CPG and its structure is retained. Basic flow-charts and decision trees [31] are commonly used by physicians. More sophisticated visualizations have been proposed, such as the representation of Asbru plans with complex temporal relations as parallel tracks in a three-dimensional perspective view [32]. Another approach for presenting decision trees consists in browsing the tree by asking to the physician questions that correspond to the various nodes of the tree (as in the GLIF browser [33] or in the OncoDoc decision support system [34]). Original interfaces have been designed to present guideline recommendations in specific medical subdomains such as antibiotherapy: R. Tsopra et al. proposed an interface presenting a short decision tree, risk factors, hospitalization criteria and antibiotic spectra [35]. Another original approach is the CPG-based quiz video game proposed by E.A. Akl et al. [36]. Simple browsers and websites [37] have also been proposed for viewing CPGs according to various models, such as GEM [38]. These simple browsers have interactive tables of content and highlighted text. Different interfaces can achieve different levels of performance [39]. M. Yasini et al. [40] proposed restructured laboratory test prescription guidelines with an interactive table of contents, a fixed set of sections and a short list of particular cases.

In this article, we proposed an original document-centric approach, using icons to tag CPG text and present lists of particular cases, which are frequent in CPGs. “Mister VCM” is used to summarize these particular cases and to classify them on the basis of anatomy and etiology. Our approach is interesting as most previous studies on CPG presentation have used flow charts and decision trees which are not well-suited for presenting particular cases. A decision tree would require a lengthy set of nodes to incorporate particular cases (for example, using questions such as “Does patient match particular case #1?”, and “If no, does patient match particular case #2?” etc.). These particular cases correspond to “conditional question” (*e.g.* “What is the management of X, given Y?”), which physicians often find it hard to answer [41].

The iconic approach we proposed can be complementary to the use of decision trees, providing icons to highlight particular cases and decision trees for presenting the main care plan.

## Conclusion

We proposed an interactive graphical user interface for facilitating physician access to CPGs, using the VCM iconic language to tag paragraphs corresponding to particular cases, and “Mister VCM” to provide a graphical summary and direct access to various paragraphs of the text. The evaluation of this interface under controlled conditions yielded promising results, the time required to find the answer to a specific clinical scenario being significantly shorter when using an interface with VCM and “Mister VCM” than when using a textual interface. Moreover, the physicians were very enthusiastic about the interactive iconic interface and this interface obtained a high SUS score (75). Future works could focus on i) a more detailed evaluation, possibly in clinical conditions, ii) the integration of VCM icons into decision trees, and iii) the automatic integration of icons and “Mister VCM” into structured guidelines (for example, in GLIF or GEM format).

## Competing interests

The VCM language is protected by an international patent awarded to Paris 13 University (EP2018620A1, WO2007131932A2). Vidal Recos is a commercial product produced by Vidal.

## Authors’ contributions

JBL, CD and AV designed the VCM iconic language and the iconic server that generates icons and “Mister VCM”. SP, S Hassler, S Hamek, CB, NL, MF, JBL, CD and AV participated in the design of the iconic user interface. SP integrated VCM icons into Vidal Reco. S Hassler, S Hamek, CB, NL, MCBZ and MF organized and analyzed the focus groups and the evaluation. JBL drafted the initial version of the manuscript. All authors read and approved the final manuscript.

## Pre-publication history

The pre-publication history for this paper can be accessed here:

http://www.biomedcentral.com/1472-6947/14/77/prepub
